# Supplementary material for: Patterns of Intron Gain and Loss in Fungi
Source: PLoS Biol. 2004 Nov 30;2(12):e422. doi: 10.1371/journal.pbio.0020422 (PMC532390; doi:10.1371/journal.pbio.0020422)
Supplement: Table S1 — Also available at http://genes.mit.edu/NielsenEtAl/. (4.3 MB ZIP). [file pbio.0020422.st001.zip › NielsenEtAl/html/616.html]

AN7479.1.NCU06457.1.MG08897.1.FG08865.1


```
 CLUSTAL W (1.82) Multiple Sequence Alignments - Introns Inserted


Sequence 1: NCU06457.1	580 aa
Sequence 2: MG08897.1	586 aa
Sequence 3: AN7479.1	574 aa
Sequence 4: FG08865.1	563 aa
Alignment Length: 595 aa
Number Identitical Residues: 245 aa
Alignment Score (without introns) 12703


MG08897.1 	MAALQELADRTKAEDNVYVDTDVGVDGNEADGSEQKPYKSLGYAFIRNHGLPTKTYQIRA
NCU06457.1	MATPQELADRTKSDAKVYVDTVAGKDDESADGSEAKPYQSLYHALIQNLDKPAPTYLTKV
FG08865.1 	MAV-------------IYIDEEAGSDLPEIQGTESAPFKSLLEAYFRHG--AESEYQVKK
AN7479.1  	MAS-------------IYIDEDAGRDDQTANGSESAPYKTLLHAFLQHDPSEGTQYLTRK
          	**              :*:*  .* *    :*:*  *:::*  * :::       *  : 

MG08897.1 	STTGALGPDEEPSTRLIWKEPAKSAVKKAQSMLDAHKKKLLKADQAAAAEEELRQKRLKN
NCU06457.1	A------PKEGSTDGPTWEEAAKSAMKKAVGRVDQYKKKLQKEKEALKKEEEERQQRLKN
FG08865.1 	K------------DDEEYKPAAKSALKKAVAYAAGQKKKLDAAAKRAEKDAQDEAARLAV
AN7479.1  	SLTEPTGEGADPESKLEWKPATKSAIKKATNLLEQRKKKAAKEQQLAIREKEEAEKRRLV
          	  : . .    .     :: .:***:***       ***     :    : :    *   

MG08897.1 	LEDAKNIKITEDPSLPPATRVELWQKD------LELG--EGDNKGPRVKVFGRIHHLRVQ
NCU06457.1	LEDAKKIVLKEDPSLPEAVRIRLNNKS------IELG--DESKKGTRVKVFGRIHRLRVQ
FG08865.1 	LEEAKKIKITNDPSLPEPVSISLHQTDSSVIGTLRKSKDEPTEGVKRVSVQGRVNRVAKQ
AN7479.1  	LEEAKKVVIKEDTSLPKPVRIRLDVTDP---AIVKLGSPESDTPGTRVRVLGRVHRLRSQ
          	**:**:: :.:*.*** .. : *  ...   . :. .. :      ** * **::::  *

MG08897.1 	KHATFLTLKSGPDQLQCIL-SGDLTKTYEALLFAQGTALFVYGELRKVLDGKNAPDGREL
NCU06457.1	KHATFLTLKDGYGFLQCVLPAGDLTKNYDALLFAQETALVLYGELKKLPAGATAPDGREL
FG08865.1 	GGLIFVTLRRGVNYMQCLL-SGDLSKTYDALTLSRETSLEIIGELWEVPAGAHAPLDREL
AN7479.1  	KDMIFLTLSDGYGYLQCVL-TGDLVKTYDAMTLTLETSIALHGEMRAVPPKQHAPNNREL
          	    *:**  * . :**:* :*** *.*:*: ::  *:: : **:  :     ** .***

MG08897.1 	QVDYYEVTGRAPGDADAIANKVSWSQDIWESDMLDNRHLVLRGDQTTSVLTVREAVELAF
NCU06457.1	QVDYYKVLGHAPSDLDAITNKVSRDQDAWDAAMLDNRHLVLRGENASALMKLREFVELAF
FG08865.1 	RADYFRVIAKAPGGDDAFTNIVPVDGD--NNTLMNLRHLTLRHEKPAAVMFVRDVVEDAF
AN7479.1  	HVDFYTIIGRAAGDKEAITTRVAHDSD--PQTLYNNRHLVLRGETASSVMKVRAATLKAF
          	:.*:: : .:*... :*::. *. . *     : : ***.** : .:::: :*  .  **

MG08897.1 	IKTFESMRIKKVSPPALVQTQVEGGSTLFKFDYYGEAAYLTQSSQLYLETVLPSLGNVYC
NCU06457.1	VDTYRELEFRKVSPPAMVQTQVEGGSTLFKYDYYGEEAYLTQSSQLYLETVLASMGQVYC
FG08865.1 	HTVYKELDIKKVSPPALVQTQVEGGSTLFKFDYYGETSYLTQSSQLYLETVLPVLGDCYC
AN7479.1  	RQTFDELRMTEVTPPAMVQTQVEGGSTLFKFDYYGEDAYLTQSSQLYLETCIPSLGDVFC
          	  .: .: : :*:***:*************:***** :************ :. :*: :*

MG08897.1 	IEKSFRAEKSLTRRH~LSEYTHVEGELDGINFEDLLVHIEEVICRVVDSVLDNPEVARHV
NCU06457.1	IEKSFRAEKSLTRRH~LSEYTHVEAELDFIEFNDLLDHLEEIISKVVDRVLNNETAAGYL
FG08865.1 	IEKSFRAEKSLTRRH0LSEYSHVEAELDFITFDNLLDHLEHVICRVIDLTLENPTAAEAI
AN7479.1  	VCPSFRAEKSLTRRH~LSEYTHIESELDFIEFTDLLDHLEAVICRVLEIVLADPVAKPII
          	:  ************ ****:*:*.*** * * :** *:* :*.:*:: .* :  .   :

MG08897.1 	KALNPDFVKPSRPFMRMKYSDAIDWLNAQDPPILNEEGHPHVFGDDI~AEAAERRMTDII
NCU06457.1	KVLNPDFKKPEK-FLRMKYEDAIEWLNKQDPPILNEEGNPHVFGDDI~AEAAERRMTDII
FG08865.1 	KKYNPDFQKPTRPFLRMRYSDAIDWLR--ERGIKNEEGNDHTFGDDI2AEAAERKMTDEI
AN7479.1  	AQLNPGFQMPSRPFLRMKYSDAIEWLR--EHEIPNEEGKPHEFGDDI~AEAAERKMTDII
          	   **.*  * :.*:**:*.***:**.  :  * ****: * ***** ******:*** *

MG08897.1 	NRPILLTHFPVEIKAFYMKKDPNDLRVTESVDVLM~PGVGEIVGGSMRMEGYEDLMAAFK
NCU06457.1	NRPILLTHFPVEIKAFYMKKDPNDLRVTESVDVLM1PGVGEIVGGSMRMDDYDELIEAYK
FG08865.1 	NRPIFLTHFPVEIKSFYMSKAKDDPRVTESVDVLM~PGVGEIVGGSMRIWDYEELMAGYK
AN7479.1  	NRPIFLTHFPAEIKAFYMKKDPEDRRVTESVDVLM~PGVGEIVGGSMRMDDWDELMAAYK
          	****:*****.***:***.*  :* ********** ************: .:::*: .:*

MG08897.1 	REGIDPAPYYWYLDQRK~YGTSPHGGYGLGLER~FLAWIAKQHTVRTCCLYPRFMGRCKP
NCU06457.1	KNHIGHEPYFWYTDQRK~YGTSPHGGYGLGLER~FLAWMANQHSVRTTCLYPRFMGRCKP
FG08865.1 	REGIDPTGYFWYTDQRK~YGSTPHGGYGLGAER2FLAWLLKLWTVREACLYPRYMGRCTP
AN7479.1  	HEGMDPSPYYWYTDQRK2YGTSPHGGYGLGLER~FLAWLCARYTVRDCSLYPRFTGRCTP
          	:: :.   *:** **** **::******** ** ****:    :**  .****: ***.*

NCU06457.1	
MG08897.1 	
AN7479.1  	
FG08865.1 	
          	
```
